# COSMOsim3D for drug-similarity, alignment, and molecular field analysis

**DOI:** 10.1186/1758-2946-4-S1-P18

**Published:** 2012-05-01

**Authors:** Andreas Klamt, Karin Wichmann, Michael Thormann

**Affiliations:** 1 COSMOlogic GmbH&CoKG, Leverkusen, and Inst. of Physical and Theoretical Chemistry, University of Regensburg, Germany; 2 Origenis GmbH, Martinsried, Germany

## 

By the practical success of the COSMO-RS fluid phase thermodynamics model [1,2] in many areas of chemistry, the COSMO polarization charge densities σ have been proven to be excellent descriptors for the quantification of the most important kinds of molecular interactions in the liquid phase, as polar interactions, hydrogen bonding and hydrophobicity. In several comparisons and blind tests COSMO-RS predictions for free energies and enthalpies of molecules in solution have been shown to be more accurate than those of most other methods in used in computational chemistry.

Since the same intermolecular interaction modes, which govern fluid phase thermodynamics, are also responsible for binding of ligands to receptors, it is most plausible that a σ-based description of of ligand-ligand similarity or of ligand-receptor interactions should be very promising. Yet, since in COSMO-RS theory the 3D- distribution of the polarization charge density σ on the molecular surface is reduced to a histogram, the σ-profile, the initial approaches in this direction [3] mostly have disregarded the spatial distribution of surface polarities. Only recently we have achieved a 3D-representation of the surface polarization charges by forming generating local σ-profiles on a regular grid. Based on their local σ-profiles, a 3D σ-similarity of two molecules can be defined as the sum of the σ-similarities on the grid points and by optimizing this similarity through rotation and translation of the probe molecule versus the target. This method is introduced here as COSMOsim3D. The quality of this COSMOsim3D similarity measure is demonstrated by a large scale evaluation on bio-isosters.

The same technique can be used to consistently align a set of ligand molecules based on their local σ-profiles. After such alignment, the local σ-profiles or the set of local σ-moments, which is a compressed representation of the σ-profiles, are available as a grid-based local descriptor set, which can be used for subsequent molecular field analysis (MFA). COSMO-RS theory even provides a rationale that the binding free energy, including desolvation, must be a linear function of this set of descriptors. First validation studies have proven that the COSMOsim3D based MFA yields very promising results.
